# Links across ecological scales: Plant biomass responses to elevated CO_2_

**DOI:** 10.1111/gcb.16351

**Published:** 2022-09-07

**Authors:** Julia Maschler, Lalasia Bialic‐Murphy, Joe Wan, Louise C. Andresen, Constantin M. Zohner, Peter B. Reich, Andreas Lüscher, Manuel K. Schneider, Christoph Müller, Gerald Moser, Jeffrey S. Dukes, Inger Kappel Schmidt, Mark C. Bilton, Kai Zhu, Thomas W. Crowther

**Affiliations:** ^1^ Institute of Integrative Biology ETH Zurich (Swiss Federal Institute of Technology) Zurich Switzerland; ^2^ Department of Earth Sciences University of Gothenburg Gothenburg Sweden; ^3^ Department of Forest Resources University of Minnesota St. Paul Minnesota USA; ^4^ Hawkesbury Institute for the Environment Western Sydney University Penrith New South Wales Australia; ^5^ Institute for Global Change Biology, and School for the Environment and Sustainability University of Michigan Ann Arbor Michigan USA; ^6^ ETH Zurich Institute of Agricultural Science Zurich Switzerland; ^7^ Agroscope, Forage Production and Grassland Systems Zurich Switzerland; ^8^ Institute of Plant Ecology Justus Liebig University Giessen Germany; ^9^ School of Biology and Environmental Science and Earth Institute University College Dublin Dublin Ireland; ^10^ Department of Forestry and Natural Resources Purdue University West Lafayette Indiana USA; ^11^ Department of Biological Sciences Purdue University West Lafayette Indiana USA; ^12^ Department of Global Ecology Carnegie Institution for Science Stanford California USA; ^13^ Geosciences and Natural Resource Management University of Copenhagen Copenhagen Denmark; ^14^ Department of Agriculture and Natural Resources Sciences Namibia University of Science and Technology (NUST) Windhoek Namibia; ^15^ Department of Environmental Studies University of California Santa Cruz California USA

**Keywords:** carbon dioxide, carbon turnover, CO_2_ fertilization, free‐air CO_2_ enrichment (FACE), global carbon cycle, plant demography, terrestrial carbon storage

## Abstract

The degree to which elevated CO_2_ concentrations (e[CO_2_]) increase the amount of carbon (C) assimilated by vegetation plays a key role in climate change. However, due to the short‐term nature of CO_2_ enrichment experiments and the lack of reconciliation between different ecological scales, the effect of e[CO_2_] on plant biomass stocks remains a major uncertainty in future climate projections. Here, we review the effect of e[CO_2_] on plant biomass across multiple levels of ecological organization, scaling from physiological responses to changes in population‐, community‐, ecosystem‐, and global‐scale dynamics. We find that evidence for a sustained biomass response to e[CO_2_] varies across ecological scales, leading to diverging conclusions about the responses of individuals, populations, communities, and ecosystems. While the distinct focus of every scale reveals new mechanisms driving biomass accumulation under e[CO_2_], none of them provides a full picture of all relevant processes. For example, while physiological evidence suggests a possible long‐term basis for increased biomass accumulation under e[CO_2_] through sustained photosynthetic stimulation, population‐scale evidence indicates that a possible e[CO_2_]‐induced increase in mortality rates might potentially outweigh the effect of increases in plant growth rates on biomass levels. Evidence at the global scale may indicate that e[CO_2_] has contributed to increased biomass cover over recent decades, but due to the difficulty to disentangle the effect of e[CO_2_] from a variety of climatic and land‐use‐related drivers of plant biomass stocks, it remains unclear whether nutrient limitations or other ecological mechanisms operating at finer scales will dampen the e[CO_2_] effect over time. By exploring these discrepancies, we identify key research gaps in our understanding of the effect of e[CO_2_] on plant biomass and highlight the need to integrate knowledge across scales of ecological organization so that large‐scale modeling can represent the finer‐scale mechanisms needed to constrain our understanding of future terrestrial C storage.

## Graphical abstract

The degree to which elevated CO₂ concentrations (e[CO₂]) increase the amount of carbon assimilated by vegetation plays a key role in climate change. Yet, it remains highly uncertain. Here, we review the effect of e[CO₂] on plant biomass across multiple levels of ecological organization, scaling from physiological responses to population‐, community‐, ecosystem‐, and global‐scale dynamics. We find that evidence for a sustained biomass response to e[CO₂] varies across ecological scales. By exploring these discrepancies, we identify gaps in our understanding of the effect of e[CO₂] on plant biomass and highlight the need to integrate knowledge across scales of ecological organization.

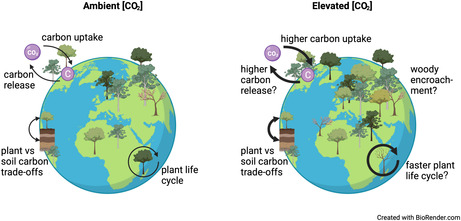

## 1 INTRODUCTION

Global atmospheric CO_2_ concentrations ([CO_2_]) have risen from ~275 ppm (MacFarling Meure et al., 2006) to ~415 ppm (National Oceanic and Atmospheric Administration (NOAA), 2021) since preindustrial times and are projected to continue increasing over the rest of this century (Prentice et al., 2001). Despite the direct impacts of elevated CO_2_ concentrations (e[CO_2_]) on the global climate (IPCC, 2014), rising concentrations might indirectly feedback to the climate system by stimulating plant productivity (Farquhar, 1997; Jarvis, 1995). Yet, the effect of e[CO_2_] on plant biomass is one of the largest uncertainties in terrestrial biogeochemical models (Huntzinger et al., 2017). Specifically, despite the shared recognition that e[CO_2_] increases local plant productivity in the short term, there is considerable disagreement about the magnitude of this effect at a global scale (Ciais et al., 2013; Terrer et al., 2019). Across nine terrestrial carbon (C) cycle models, the estimated increase in biomass for the period 1980–2010 ranged from 5% to 27% per 100 ppm CO_2_ (Terrer et al., 2019). However, an emerging body of evidence also suggests that these increases in plant biomass may potentially be more transient than previously expected, and that the plant response to e[CO_2_] may be limited by physiological, population‐, community‐, and ecosystem‐level dynamics under future [CO_2_] (Bugmann & Bigler, 2011; Körner, 2004; Norby et al., 2010). Given the importance of the plant biomass response to e[CO_2_] for future climate projections, determining the magnitude and duration of this potential C cycle feedback is critical for improving the accuracy of future climate change scenarios (Andresen et al., 2016).

To date, most of our understanding of the e[CO_2_] effect stems from short‐term studies of only a few years. Due to the high costs associated with CO_2_ enrichment studies, only a few of them offer insights into the effect of e[CO_2_] on plants after a decade or more (Liebermann et al., 2019; McCarthy et al., 2010; Reich et al., 2018; Schneider et al., 2004; Talhelm et al., 2014). Relying on short‐term CO_2_ experiments to project climate–vegetation feedbacks, Earth system models (ESMs) predict that e[CO_2_] stimulate plant productivity (and associated C stocks) and thereby counteract future increases in atmospheric [CO_2_] (Huntingford et al., 2013; Sitch et al., 2008). However, these inferences from short‐term studies do not necessarily capture some of the changes in plant physiology (e.g., acclimation), population (e.g., mortality), community (e.g., competition patterns), and ecosystem ecology (e.g., soil nutrient dynamics) that have the potential to constrain the effect of e[CO_2_] on plant biomass over time (Walker et al., 2021). Using short‐term responses to infer long‐term outcomes under e[CO_2_] may be particularly misleading for ecosystems with long‐lived species with slow generational turnover, in which it can take decades to reach new equilibrium dynamics following environmental change. The lack of reconciliation of research across different levels of ecological organization, which ranges from leaf‐level gas exchange measurements to global‐scale modeling studies, represents a major hurdle for our confidence in C cycle projections.

**FIGURE 1:**
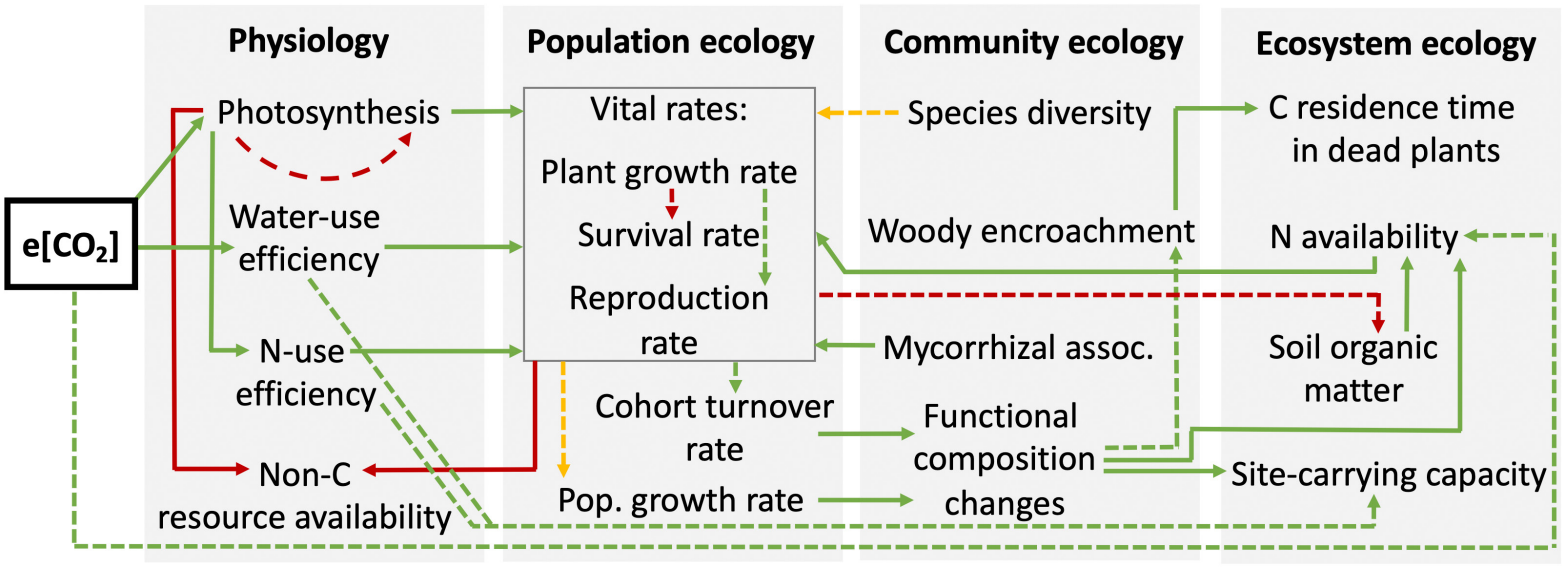
Conceptual diagram of the effects of e[CO_2_] on plant biomass accumulation across scales as discussed in this review. The directionality of effects is indicated by the color of arrows (green = positive, red = negative, yellow = unclear). The confidence in this direction is indicated by line type (solid = high confidence, dashed = low confidence). As global‐scale ecology is based on the extrapolation or observation of smaller‐scale processes, we excluded it from this figure.

Here, we review the current state of knowledge of the direct effects of e[CO_2_] on plant biomass accumulation to synthesize and compare insights across the different levels of ecological organization, scaling from (i) physiological responses to changes in (ii) population, (iii) community, (iv) and ecosystem responses (Figure 1). We then consider these mechanisms in the context of global‐scale observations. Although e[CO_2_] influences plant biomass in concert with other changes in climate and atmospheric composition, this review primarily focuses on direct consequences of e[CO_2_] on plants, that is, topics like the effects of global warming due to e[CO_2_] are not discussed. Within each section of the review, we highlight when responses observed in short‐term studies may not necessarily translate to long‐term outcomes. To do this, we compare insights from CO_2_ enrichment experiments, natural CO_2_ springs, tree ring observational studies as well as satellite imagery and discuss implications for global biochemical modeling and C cycle projections. Previous work has highlighted the ecological mechanisms governing the effect of e[CO_2_] on plant biomass (Walker et al., 2021). We build on this work by exploring how evidence for plant biomass responses to e[CO_2_] varies across different levels of ecological organization across individuals, populations, communities, and ecosystems. This systematic approach allows us to identify the mechanisms where we have high confidence, and those that require further research attention. By highlighting commonalities and discrepancies among these ecological scales, we aim to motivate efforts to align perspectives in order to improve the accuracy of future climate change projections.

## 2 PHYSIOLOGY

Plant C assimilation is a fundamental flux in the C cycle, which is why an e[CO_2_]‐induced stimulation of photosynthesis can create a powerful negative climate feedback with plant C. In this section, we discuss the mechanisms by which e[CO_2_] can improve both water‐ and nutrient‐use efficiency as well as the consequences for photosynthesis. In addition, we outline the perspective for a long‐term effect of e[CO_2_] on plant C assimilation in natural, commonly nutrient‐limited ecosystems.

### 2.1 Elevated [CO_2_ ] increase water‐use efficiency and carboxylation rates

Rising atmospheric [CO_2_] have the potential to increase photosynthetic rates. Whether this translates to higher plant growth depends on the extent to which the additional C is used for respiration and biomass production. At higher [CO_2_], there is a lower chance of the energetically costly process of photorespiration in C_3_ plants, which occurs when Rubisco uses O_2_ instead of CO_2_ as a substrate (Drake et al., 1997; Long et al., 2004). As the O_2_ affinity of Rubisco increases with rising temperatures, e[CO_2_] might have a particular strong effect on C_3_ plants in warm climates (Jordan & Ogren, 1984). In addition to decreasing photorespiration, e[CO_2_] alleviates substrate limitation of Rubisco at current [CO_2_], which causes higher carboxylation rates (Drake et al., 1997; Long et al., 2004). Yet, with increasing [CO_2_], C_3_ plants seem to shift from being mainly Rubisco‐limited (*V*_cmax_‐limited) toward being mainly limited by the capacity for the regeneration of Rubisco's acceptor molecule ribulose‐1,5‐bisphosphate (*J*_max_ limitation) (Ainsworth & Rogers, 2007; Long & Bernacchi, 2003). This suggests that the effect of higher carboxylation rates might decrease at high [CO_2_], and further increases in [CO_2_] should eventually lead to a saturation of the photosynthetic response to e[CO_2_] (Ainsworth & Rogers, 2007; Long & Bernacchi, 2003). Yet, while this saturation point remains unclear, increases in [CO_2_] in the magnitude projected for the next decades to centuries can be expected to translate to higher photosynthesis unless other key resources needed to maintain C fixation and plant growth become limiting.

The effects of e[CO_2_] on photosynthesis stem from the change in operational capacity of the photosynthetic enzyme Rubisco and a change in stomatal conductance. In a trade‐off between reducing water loss and absorbing CO_2_ for photosynthesis, plants regulate the openness of their stomata. As atmospheric [CO_2_] rise, increased stomatal closure allows plants to maintain the same rate of C absorption at a lower water loss. In line with this, decreased stomatal conductance has been reported from both short‐ and long‐term Free‐Air CO_2_‐enrichment (FACE) studies, as well as observations at natural CO_2_ springs, which indicates that the effect of e[CO_2_] on stomatal conductance may persist over time (Ainsworth & Rogers, 2007; Pastore et al., 2019; Saban et al., 2019). The resulting positive effect of e[CO_2_] on leaf‐level water‐use efficiency and/or intrinsic water‐use efficiency (hereafter: WUE) is widely acknowledged (Ainsworth & Rogers, 2007) and in line with evidence from eddy‐covariance and observational tree ring research. These studies have shown positive correlations between increased WUE and rising [CO_2_] over the past decades to 150 years (Dekker et al., 2016; Keenan et al., 2013; Mastrotheodoros et al., 2017; Peñuelas et al., 2011; Silva & Anand, 2013; van der Sleen et al., 2014), yet with very different magnitudes in their estimates (Lavergne et al., 2019) as also discussed by Walker et al. (2021). Together, this suggests that e[CO_2_] might support higher plant growth rates in water‐limited ecosystems (Blumenthal et al., 2013) and shift species composition toward a higher abundance of more water‐demanding species relative to more drought‐resistant species (Körner, 2003).

### 2.2 Carbon source–sink dynamics limit the photosynthetic response to e[CO_2_ ]

The positive effect of e[CO_2_] on photosynthesis can decrease through a progressive downregulation of photosynthetic capacity (photosynthetic acclimation). In CO_2_ enrichment studies (Ainsworth & Long, 2004), this manifests in a decrease in the maximum carboxylation rate (*V*_cmax_) and the maximum rate of electron transport (*J*_max_), which are the two main proxies of photosynthetic capacity. Therefore, despite the fact that e[CO_2_] of ~565 ppm are commonly observed to lead to an instantaneous increase in net photosynthesis of ~40% to ~65% in C_3_ species (Bader et al., 2010; Ellsworth, 1999; Lee et al., 2001), acclimation often causes photosynthetic stimulation to drop below the immediate response, to long‐term increases ranging from close to zero to ~45% relative to ambient conditions (Ainsworth & Long, 2004; Bader et al., 2010; Pastore et al., 2019; Saban et al., 2019; Warren et al., 2015).

While there is ample evidence for this progressive photosynthetic acclimation, which may happen within a few months (Lee et al., 2001) or develop gradually over the course of years (Norby et al., 2010), and persist for decades (Pastore et al., 2019), the mechanisms responsible for this dampening of the photosynthetic response are not well understood. However, acclimation is commonly assumed to be driven by a gradual build‐up of C sink limitations whereby, upon a lack of plant growth‐limiting non‐C resources, the demand for C compounds from photosynthesis decreases relative to the supply (C sink limitation). In this context, the build‐up of photosynthates can lead to a reduction in the levels of the nitrogen‐rich photosynthetic enzyme Rubisco (Ainsworth & Long, 2004; Ainsworth & Rogers, 2007). While the decrease in nitrogen (N) levels has traditionally been seen as a primarily limitation‐driven dilution effect (Stitt & Krapp, 1999), more recent research suggests that acclimation may be a result of altered trait investment under resource limitation (Smith & Keenan, 2020). In response to altered environmental conditions (e.g., e[CO_2_]), plants may adjust their photosynthetic machinery over time to maintain the highest rate of C fixation in a given environment at the lowest possible cost (resource‐use optimization; Smith & Keenan, 2020; Wright et al., 2003). In line with this, lower Rubisco levels may create the opportunity for trait investment to optimize the use efficiency of other productivity‐limiting resources, thereby potentially alleviating C sink limitations (Smith & Keenan, 2020). However, lower Rubisco levels can also dampen the e[CO_2_]‐induced photosynthetic stimulation. Whether or not this affects C assimilation depends on the degree of enzyme reduction as well as if the plant's photosynthesis is limited by Rubisco or the regeneration of the Rubisco acceptor molecule at e[CO_2_] (Ainsworth & Rogers, 2007; Smith & Keenan, 2020).

Within the e[CO_2_] literature, a low availability of soil N and the immobilization of N in a larger body of standing biomass (progressive nitrogen limitation, PNL; Comins & McMurtrie, 1993; Luo et al., 2004) are by far the most commonly discussed constraint on photosynthetic stimulation and plant growth (Ainsworth & Long, 2004; Kirschbaum, 2011; Stitt & Krapp, 1999; Wang & Wang, 2021). While other resources limiting photosynthesis and plant growth might also cause photosynthetic acclimation to e[CO_2_], they have received much less attention. For example, phosphorus (P) has not been a strong research focus in the past, and CO_2_ enrichment studies have rarely manipulated soil P levels and are scarce in P‐limited, tropical ecosystems (Du et al., 2020). Yet, there seems to be an increasing awareness of the relevance of P for the effect of e[CO_2_] on plant biomass (Ellsworth et al., 2017; Jiang, Caldararu, et al., 2020; Jiang, Medlyn, et al., 2020; Tissue et al., 2010) and it can be expected that a wider focus on other plant resources will underline the importance of different non‐N resources for photosynthetic acclimation to e[CO_2_].

In addition to resource limitations, e[CO_2_] can induce shifts in C source–sink dynamics that can affect the duration of plant C assimilation activity throughout the growing season and the timing of autumn leaf senescence. In herbaceous plants, a decrease in the C sink through nutrient depletion or a prevention of seed formation has been linked to earlier leaf senescence (Guitman et al., 1991; Kumar et al., 2019). While the relationship of tree C source–sink dynamics and leaf senescence has received less attention than herbaceous plants, there are indications that a higher C source activity in trees (e.g., due to more leaves or earlier leaf‐out) can be followed by earlier autumn leaf senescence (Fu et al., 2014; Zani et al., 2020). However, delayed or unchanged senescence under e[CO_2_] from multiple CO_2_ enrichment studies (Herrick & Thomas, 2003; Norby et al., 2003; Taylor et al., 2008) provides alternative expectations (Norby, 2021; Zani et al., 2021). If the C source–sink balance proves to be a general driver of autumnal leaf senescence, it will be even more important for calculations of future terrestrial C fluxes to not only consider exogenous factors like nutrient or water availability. In addition, the inclusion of plant‐internal determinants of C sink capacity, such as the life stage, could add important information for improving the accuracy of model predictions (Zani et al., 2020).

## 3 POPULATION ECOLOGY

Higher photosynthetic rates in plants under e[CO_2_] can increase plant growth if the availability of non‐C resources permits. However, stimulated plant growth and increased resource demands can also induce trade‐offs in the allocation of limiting resources needed to maintain vital rates, that is, survival, growth, and reproduction (Stearns, 1989). In addition to these life‐history trade‐offs, self‐thinning is expected to affect population growth dynamics in natural ecosystems (Reineke, 1933). In this section, we discuss how plant growth under e[CO_2_] and possibly associated changes in resource allocation can influence plant population dynamics in a high CO_2_ environment.

### 3.1 Plant growth response to e[CO_2_ ] depends on non‐C resources

The increase in realized photosynthesis under e[CO_2_] provides a base for potential plant growth stimulation. However, the fact that photosynthesis of most plants acclimates to e[CO_2_], which is frequently assumed to be a consequence of N limitations, indicates that non‐C resource limitations might dampen e[CO_2_]‐induced plant growth stimulation over time. Indeed, while CO_2_ enrichment studies commonly show an increase in plant growth rates under e[CO_2_] (Gebauer et al., 1996; Kimball et al., 2007; Norby et al., 1995; Peltola et al., 2002; Wang & Wang, 2021), there are also indications from such experiments that that the positive effect of e[CO_2_] might not persist over time in low N (Gebauer et al., 1996; Norby et al., 2010; Peltola et al., 2002) and P conditions (Ellsworth et al., 2017). Also, while the vast majority of CO_2_ enrichment tree studies focuses on young individuals in their most productive and responsive demographic phase, the effect of e[CO_2_] on plant growth rates is less clear for later life stages (Bader et al., 2010; Ellsworth et al., 2017). In observational tree ring studies (Battipaglia et al., 2015; Giguère‐Croteau et al., 2019; Peñuelas et al., 2011; Silva & Anand, 2013; van der Sleen et al., 2014), estimates of plant growth rates varied greatly, with many studies even showing negative trends under historical increases in [CO_2_]. This argues against e[CO_2_] as a driver of increased tree growth or for an offset of a positive effect of e[CO_2_] through counteracting factors like increases in temperature (Battipaglia et al., 2015; Silva & Anand, 2013) or nutrient limitation (Giguère‐Croteau et al., 2019; Peñuelas et al., 2011; Silva & Anand, 2013 ). Therefore, evidence from both tree ring and CO_2_ enrichment studies indicates that e[CO_2_]‐induced plant growth stimulation might dampen over time due to resource limitations.

### 3.2 Plant survival rates may decrease under e[CO_2_ ]

**FIGURE 2:**
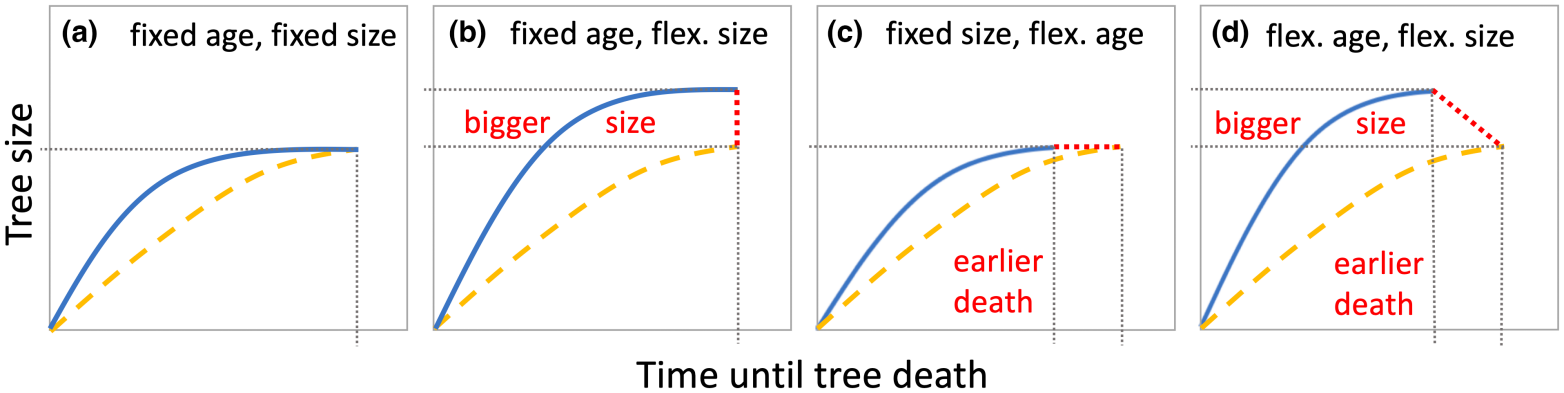
Conceptual diagram of the effects of increased tree growth rates on tree survival. There are four possible scenarios with varying consequences for C sequestration in tree biomass. (a) Tree death occurs at a fixed age while tree size is capped, (b) death at fixed age but tree size is flexible, (c) death at fixed size but age is flexible, (d) death at flexible age and size. The blue (solid) and yellow (dashed) curves depict biomass under e[CO_2_] and ambient [CO_2_]. The red lines (dotted) and text indicate consequences for C sequestration at the time of tree death. Figure redrawn and modified from Büntgen et al. (2019).

Global models differ considerably in their incorporation of mortality (Pugh et al., 2020), explaining why model uncertainties around plant C residence time are bigger than those around NPP projections (Friend et al., 2014). The observed increase in plant growth rates under e[CO_2_] does not necessarily alter long‐term C storage in plant biomass if increased plant growth under e[CO_2_] is counterbalanced by reduced survival and plant longevity (Figure 2; Körner, 2017). Unfortunately, CO_2_ enrichment studies with perennial species have not continued long enough to capture changes in survival patterns across all life stages which is why our understanding of the effects of e[CO_2_] on survival is primarily limited to early life stages. In agricultural CO_2_ enrichment studies, annual crops showed earlier leaf senescence under e[CO_2_] (Kimball et al., 1995; Sakai et al., 2001), but this might merely be a result of earlier C sink exhaustion under e[CO_2_] and not be an indication of decreased longevity in perennial species (see Section 2.2).

The anticipated negative effect of e[CO_2_] on plant survival is based on the widely established concept of interspecific survival–growth trade‐offs (Bigler & Veblen, 2009; Brienen et al., 2020; Bugmann & Bigler, 2011). Since survival–growth trade‐offs may also occur at the intraspecific level (Brienen et al., 2020; Negreiros et al., 2014; Russo et al., 2021; Seiwa, 2007), theory suggests that higher plant growth rates under e[CO_2_] should go hand in hand with a higher mortality risk (McDowell et al., 2018, 2020). There are various intrinsic and extrinsic mechanisms that might interactively drive this negative correlation between growth and survival, including increased rates of wind damage as a result of reductions in wood density and mechanical strength, and a lower investment in antiherbivore defenses (Barnett & Jeronimidis, 2003; Bugmann & Bigler, 2011; Coley et al., 1985; King et al., 2005; Larson, 2001). In addition, irrespective of the mechanisms driving survival–growth trade‐offs, a larger plant body can also translate to a higher susceptibility to environmentally induced risk factors, for example, through an increased risk for hydraulic failure as well as fewer options for downregulation in times of stress (Arendt, 2010; Bigler & Veblen, 2009; McDowell et al., 2008). In line with this, a modeling study using observational survival–growth relationships from tree‐ring data suggested that tree growth rate stimulations in the magnitude of those observed under e[CO_2_] are associated with a reduction in longevity, which might ultimately bring the net effect of e[CO_2_] on standing biomass close to zero (Bugmann & Bigler, 2011). This suggests that e[CO_2_]‐induced increases in plant growth might be counterbalanced by reduced survival and plant longevity (i.e., faster cohort turnover).

Unrelated to survival–growth trade‐offs, increased plant growth under e[CO_2_] can intensify the extent of competitive exclusion among plants as they develop. Ultimately, because there is a finite amount of resources (including space, light, nutrients, etc.) in any location, it is necessary that the number of plants falls as the size of those plants increases (Reineke, 1933). Given this negative relationship, e[CO_2_]‐induced increases in plant growth can intensify competition for limiting resources. If the shape of the negative proportional relationship between plant number and plant volume (self‐thinning curve) is unaffected by e[CO_2_], the final stand volume resulting from both slow and fast growth will be identical, even though e[CO_2_]‐induced faster plant growth can speed up the rate by which this final state is reached. Experiments have shown that site quality can affect self‐thinning curves (Ge et al., 2017) and e[CO_2_] could improve site quality via effects on resource‐use efficiency or resource availability. For example, e[CO_2_] has a positive effect on both WUE (Ainsworth & Rogers, 2007; Pastore et al., 2019; see Section 2.1) and N‐use efficiency (Drake et al., 1997; Stitt & Krapp, 1999), which implies that e[CO_2_] might change self‐thinning curves. Supporting evidence comes from the only study we found that investigated this matter directly, which suggests that e[CO_2_] might be able to sustain a higher stand basal area at an equivalent tree number per area (Kubiske et al., 2019). Therefore, it is possible that changing self‐thinning curves under e[CO_2_] allow for a higher stand volume and accordingly higher plant biomass levels in the long term, yet more research is needed. In general, it seems that inferring long‐term standing biomass merely from e[CO_2_]‐induced increases in (short term) plant growth rates is problematic, particularly for long‐lived species with slow generational turnover, and the consideration of self‐thinning dynamics is essential for a meaningful estimate of vegetation levels under e[CO_2_].

### 3.3 Faster growth under e[CO_2_ ] seems to be associated with earlier reproductive maturity

Apart from the impacts of e[CO_2_] on survival and growth, the effect of e[CO_2_] on plant populations is shaped by reproduction dynamics. As e[CO_2_] increases the C source, more C should be available not only for plant growth but also for energetically costly reproduction (Taiz & Zeiger, 2010), leading to the expectation that absolute C investment in reproduction increases under e[CO_2_]. Indeed, evidence from a meta‐analysis suggests that herbaceous plants and shrubs under e[CO_2_] might have a higher number of flowers, fruits, and seeds as well as greater individual and total seed mass (Jablonski et al., 2002). Those few tree studies that captured the mature life stage showed that trees under e[CO_2_] also had more or heavier seeds, cones, or flowers (Darbah et al., 2008; LaDeau & Clark, 2006; Stiling et al., 2004), higher germination rates (Darbah et al., 2008), and reached maturity earlier (LaDeau & Clark, 2001). The fact that observed increases in reproductive output at e[CO_2_] coincided with an overall increase in plant growth rates (Darbah et al., 2008; Stiling et al., 2004) implies that these trends might have been the result of a faster lifecycle under e[CO_2_], in which case a plant's cumulative reproductive output over its lifespan may not change.

While higher C fixation under e[CO_2_] increases the absolute amount of energy that can be invested in reproduction, it is unclear whether and how the investment of C in reproduction relative to vegetative growth is affected. If reproductive allocation changes under e[CO_2_], this could affect the total reproductive output of a plant over its lifetime. The only study we found that measured reproductive allocation in trees under e[CO_2_] reported that trees reached maturity at a smaller size and had a higher proportional allocation to reproduction (LaDeau & Clark, 2001; Way et al., 2010). This suggests that more freely available C might enable young trees to invest more in energetically costly reproduction. However, in a meta‐analysis with herbaceous plants, e[CO_2_] was not found to increase overall reproductive allocation. Upon differentiation by reproductive strategies and functional groups, no effect of e[CO_2_] on reproductive investment was found for perennial wild species, annual or biennial plants, while perennial crops under e[CO_2_] showed a significant increase in reproductive allocation (Wang et al., 2015). Yet, this effect was likely caused by the fact that the reproductive structures of the specific crop species had been bred to a large size and thereby constituted strong C sinks (Wang et al., 2015). Therefore, it seems that the reproductive investment of herbaceous species in natural ecosystems might not be affected by e[CO_2_]. However, there is a need for more research that links lifecycle dynamics of other plant types in natural ecosystems, particularly trees, to examine reproductive allocation patterns across all life stages.

### 3.4 Effect of e[CO_2_ ] on population fitness is uncertain

Research on e[CO_2_]‐induced changes in plant performance suggests highly uncertain effects of e[CO_2_] on plant survival, growth, and reproduction rates (McDowell et al., 2020). As e[CO_2_]‐induced changes in each vital rate can have counteracting effects on plant population growth, it is critical to incorporate these complexities when evaluating the magnitude of e[CO_2_]‐driven plant biomass accumulation over time. To our knowledge, only one study has examined the effects of e[CO_2_] on all vital rates and resulting changes in population growth. Interestingly, this study reports that e[CO_2_] had pronounced, and often opposing, effects on the survival, growth, and reproduction of four grassland species (Williams et al., 2007). The effect of these changes in plant vital rates on population fitness (i.e., population growth) ranged from positive to negative (Williams et al., 2007). These findings illustrate that caution should be taken when using isolated components of plant performance (e.g., plant growth) to infer the effects of e[CO_2_] on long‐term population growth and C sequestration. Opposite effects of e[CO_2_] on vital rates can cancel each other out, resulting in a faster cohort turnover but no change in species abundance and thereby C storage over time.

## 4 COMMUNITY ECOLOGY

Interspecific variability in the responsiveness to e[CO_2_] can influence species competition and shift the equilibrium toward more responsive species (Blumenthal et al., 2013). This can have important implications for the residence time of C within a community as different functional types and species vary in their growth, C storage, and nutrient dynamics (Körner, 2017; Ruesch & Gibbs, 2008). In the following section, we discuss how community dynamics might change under e[CO_2_] and how the resulting species and functional type compositions may affect the magnitude and duration of vegetation C storage.

### 4.1 Access to non‐C resources affects plant biomass responses to e[CO_2_ ]

**FIGURE 3:**
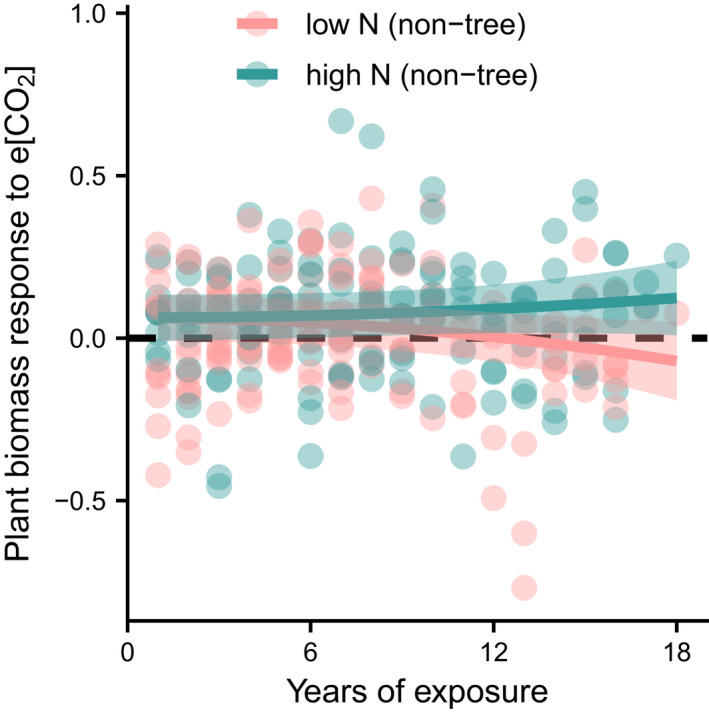
Effect of N treatment on the plant biomass response to e[CO_2_] over time. Plant biomass response to e[CO_2_] is defined as ln(aboveground standing biomass under e[CO_2_])−ln(aboveground standing biomass under ambient [CO_2_]) in six non‐tree FACE studies. We added predictions and approximated 95% confidence intervals from model‐based bootstrapping for a linear mixed‐effects model of the plant biomass response to e[CO_2_]. The main effects (time^2^, a nitrogen integer multiplied by time^2^) in the model were significant at the 5% level. For details on model structure, model diagnostics and data structure, we refer to Methods S1 and Supplementary Figures. Data (Maschler et al., 2022) adapted from Andresen et al. (2016).

As plants under e[CO_2_] become increasingly limited by non‐C resources, species that are the most successful at harvesting such resources are expected to grow faster and accumulate the most biomass, thereby outcompeting other plants and eventually becoming more dominant. Since N is considered one of the most limiting non‐C resources in natural environments (Du et al., 2020; LeBauer & Treseder, 2008), N availability is expected to be a primary factor determining the effect of e[CO_2_] on plant biomass levels over time. Consistent with this expectation, we found that the positive effect of e[CO_2_] on standing plant biomass was only sustained under high N across six non‐tree FACE studies over a span of 18 years (Figure 3). In two FACE studies with trees, N availability was either found to be a strong determinant of the spatial variation in productivity patterns (McCarthy et al., 2010), or N limitation emerged as the most likely reason for a strong decrease in e[CO_2_]‐induced NPP enhancements over time (Norby et al., 2010). Alongside other evidence (Wang & Wang, 2021), these findings underline the high importance of N availability for the response of plant biomass to e[CO_2_], thereby suggesting that any factor that increases the availability or uptake of N has the potential to impact species biomass accumulation and competitive ability under e[CO_2_].

One factor that has received a lot of attention in altering plant resource uptake and competitive ability under e[CO_2_] is the association of plants with nutritional fungal symbionts. Experimental evidence suggests that ectomycorrhizal fungi (EMF) can increase soil N availability and plant N uptake, for example, through the production of extracellular enzymes degrading organic N compounds in the soil (Abuzinadah & Read, 1986; Bending & Read, 1995), while arbuscular mycorrhizal fungi (AMF) have been shown to alleviate plant P limitation (Johnson et al., 2015; Mei et al., 2019). In line with the expectation that N‐limited plants should benefit from an association with EMF, a meta‐analysis of 83 CO_2_ enrichment studies has shown that e[CO_2_] stimulated biomass accumulation only in EMF‐ but not AMF‐associated plants under N limitation (Terrer et al., 2016). These results suggest that mycorrhizal associations are an important factor governing plant biomass responses to e[CO_2_] and therefore affect competitive structures in mixed communities. Because of the shift from relative P to N limitation toward cold regions (Du et al., 2020), EMF‐associated plants might have a competitive advantage in high‐latitude areas, whereas AMF‐associated plants may be favored in the tropics.

Besides mycorrhizal associations, there are other symbioses that can help plants acquire nutrients. For example, a bacterial symbiosis makes the growth of N‐fixing plants less dependent on soil N availability (Ainsworth & Long, 2004; Mohan et al., 2007) and, accordingly, e[CO_2_] strongly stimulated aboveground dry matter production of FACE legumes (20%; Ainsworth & Long, 2004). Furthermore, CO_2_ enrichment has been observed to increase the dominance of N fixers in a mixed forest understory community (Norby & Zak, 2011) as well as in two grassland systems (Lüscher et al., 2004; Newton et al., 2010). The positive effect of e[CO_2_] on the growth of N fixers can benefit the whole ecosystem. N fixation rates are strongly stimulated under e[CO_2_] (Liang et al., 2016), which was reported to increase soil N availability and N levels in surrounding non‐N fixers (Lee et al., 2003; Zanetti et al., 1996). If e[CO_2_]‐induced changes in N dynamics increase the productivity of non‐N fixers in the ecosystem (Brookshire et al., 2019), this might increase overall vegetation levels. Aside from temperate forests and wet savannas, it is unproductive ecosystems like arid shrublands and tropical savannas where N fixation is most common (Cleveland et al., 1999), which implies that these regions might show particularly strong relative biomass responses to e[CO_2_].

### 4.2 Woody plants may benefit the most from e[CO_2_ ]

Plant functional types differ considerably in their C storage capacity and duration. Therefore, it is key to incorporate any e[CO_2_]‐induced changes in functional group composition into global biogeochemical model predictions that focus on plant C storage dynamics. For example, the benefits of C_4_ photosynthesis in warm and arid environments, which enables C_4_ plants to actively concentrate CO_2_ around Rubisco, overlap with those resulting from e[CO_2_]. This explains why photosynthesis of C_3_ plants commonly responds more strongly to e[CO_2_] compared to C_4_ plants (Ainsworth & Long, 2004; Wang et al., 2012) and indicates reduced competitiveness of C_4_ photosynthesis under e[CO_2_] (Scott & Smith, 2022). Similarly, CO_2_ enrichment studies commonly show that C_3_ plant biomass responds more to e[CO_2_] than C_4_ biomass does (Ainsworth & Long, 2004; Curtis et al., 1999). Interestingly, recent evidence from monocultures and mixed communities suggests that progressive N shortage may potentially reverse these expected patterns of higher biomass responses in C_3_ versus C_4_ plants after years or even decades (Reich et al., 2018), but the generalizability of this phenomenon across different ecosystem types remains unclear.

**FIGURE 4:**
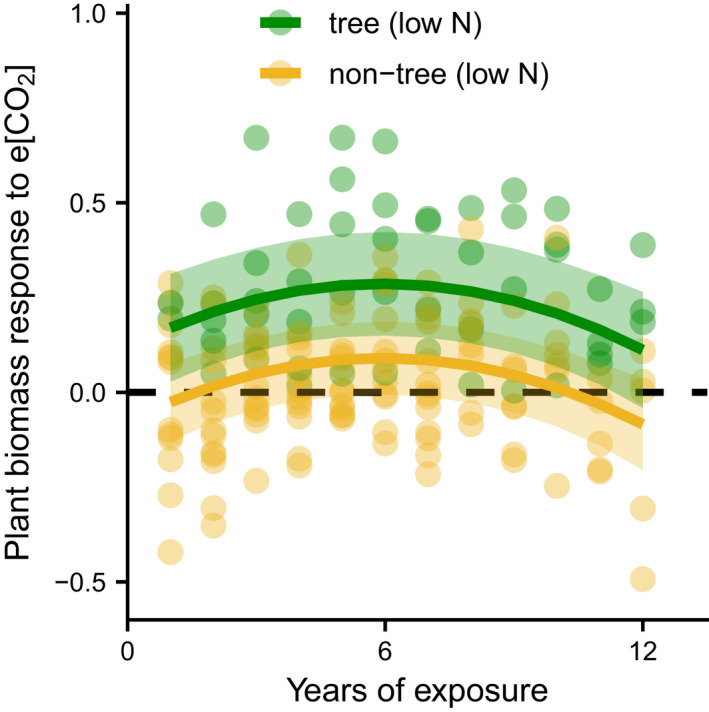
Effect of functional type on the plant biomass response to e[CO_2_] over time. Plant biomass response to e[CO_2_] is defined as ln(aboveground biomass under e[CO_2_])−ln(aboveground biomass under ambient [CO_2_]), where aboveground biomass is aboveground standing biomass for non‐trees and a measure of annual aboveground biomass increment for trees in eight FACE studies. We added predictions and approximated 95% confidence intervals for a linear mixed‐effects model of the plant biomass response to e[CO_2_]. Two of the three main effects (time, time^2^) were significant at the 5% level, while functional type was marginally significant (*p* = .051). For details on model structure, model diagnostics and data structure, we refer to Methods S1 and Supplementary Figures. Data (Maschler et al., 2022) adapted from Andresen et al. (2016).

Besides possible differences in the effect of e[CO_2_] on C_3_ versus C_4_ plants, the responsiveness of different plant functional types might also vary. For example, trees grown under e[CO_2_] in CO_2_ enrichment studies tend to show the strongest photosynthetic response to e[CO_2_] relative to other plant functional groups (Ainsworth & Rogers, 2007). Similar to photosynthetic patterns, trees tend to show a higher increase in aboveground plant biomass than other functional groups under e[CO_2_] (Ainsworth & Long, 2004; Terrer et al., 2019). Modeling how the functional type controls the effect of e[CO_2_] on aboveground plant biomass in eight FACE studies, we found that the effect of e[CO_2_] was higher in tree vs non‐tree studies (*p* = .051; Figure 4). These physiological responses are supported by an 11‐year community‐level study, which found that e[CO_2_] accelerated the successional development of an understory plant community (Souza et al., 2010). While under ambient conditions, herbaceous species consistently contributed the most to total understory biomass, there was a shift under e[CO_2_] toward a community where woody species contributed the most (Souza et al., 2010). As woody tissues have a longer C turnover time than non‐woody plant tissues, such changes could affect community C turnover dynamics. However, the importance of such trends in forest FACE studies might decline once canopy closure occurs in the (usually) young study systems and the amount of total understory biomass decreases (Bandeff et al., 2006). Also, evidence from (short‐term) CO_2_ enrichment studies with trees should be interpreted with caution because the long lifespans of trees mean that most studies have focused on early life stages that tend to be more responsive, productive, and mainly shaped by only one vital rate (plant growth), whereas this is not the case for studies using grasses or forbs.

There is some evidence that increases in [CO_2_] over the past decades might already have induced a shift toward woodier plant communities. For example, satellite imagery showed that woody cover over sub‐Saharan Africa increased by 8% between 1986 and 2016 (Venter et al., 2018) and, indeed, evidence based on experimental and observational data suggests that e[CO_2_] might have contributed to woody encroachment into water‐limited savanna ecosystems in Africa and Australia (Buitenwerf et al., 2012; Stevens et al., 2017). This is in line with the expectation that the higher biomass accumulation rates of young savanna trees (C_3_) can decrease the time it takes to reach a large enough size to withstand fire damage (Higgins & Scheiter, 2012), while C_4_ grass biomass might not respond considerably to e[CO_2_] (Ainsworth & Long, 2004; Curtis et al., 1999). In addition, higher WUE under e[CO_2_] is commonly assumed to benefit water‐demanding species (Stevens et al., 2017). Yet, other observational data from African savannas suggest that increased abundance of woody species are within their historic ranges (Zhou et al., 2021). This geographic constraint suggests that e[CO_2_] may not increase WUE to a degree where it underpins woody encroachment trends (Zhou et al., 2021). Instead, land‐use changes (Van Langevelde et al., 2003; Zhou et al., 2021) and other global change factors, such as N deposition (Köchy & Wilson, 2001; Wigley et al., 2010), have been suggested as additional or alternative drivers of woody encroachment into grasslands. An integration of such disagreements between observational studies could be facilitated by strongly needed evidence from experiments with an explicit focus on competitive patterns within mixed communities of woody C_3_ and non‐woody C_4_ species under e[CO_2_]. Since the C turnover time is longer in woody vs non‐woody biomass, a shift in community composition toward C_3_ species could benefit C sequestration irrespective of total plant biomass trends in savanna ecosystems. Given that savannas contribute approximately a third of terrestrial NPP (Grace et al., 2006), such changes in vegetation cover could have pronounced effects on the residence time of plant C at the global scale.

### 4.3 Effect of species diversity on biomass accumulation under e[CO_2_ ] is unclear

The magnitude of e[CO_2_]‐induced plant biomass accumulation at the community level is highly dependent on how efficient the community members are at harvesting non‐C resources. In line with the higher niche differentiation in multi‐ compared to single‐species communities, species and functional group richness increased e[CO_2_]‐induced biomass accumulation at the plot level in a grassland study (Reich et al., 2001, 2004). However, a meta‐analysis using ~1700 and ~500 observations of single‐ and multi‐species assemblages suggests that this intuitive assumption might neglect other important factors. Surprisingly, the average e[CO_2_]‐induced increase in total plant biomass was only 13% in studies with multiple species compared to 30% in single‐species setups (Wang, 2007). The authors suggested that this unexpected result could have been caused by high size heterogeneity in mixed‐species communities and resource usurpation by large individuals with a weak growth response to e[CO_2_]. Alternative possible explanations include an uneven representation of early‐stage versus mature growth responses, higher average [CO_2_] for the eCO_2_ treatment in single‐species studies, or the fact that species with different traits might have been selected for each study. Interestingly, in the meta‐analysis, e[CO_2_]‐induced biomass accumulation in single‐ compared to multi‐species setups was only significantly different when N was added. This finding indicates that a possibly negative effect of species diversity on e[CO_2_]‐induced biomass accumulation is mediated by N availability (Reich et al., 2001, 2004). Therefore, mixed results among niche differentiation studies under e[CO_2_] highlight the need for future research to not only focus on the effect of species richness on the plant biomass response to e[CO_2_], but also on how this relationship is mediated by resource availability. Once we have more knowledge about the mechanism behind the interplay of these factors, the incorporation of the relationship between community diversity and plant biomass responses to e[CO_2_] could improve how ESMs capture regional differences in biomass trends under e[CO_2_].

## 5 ECOSYSTEM ECOLOGY

The availability of resources and thereby the ability of plants to accumulate additional biomass under e[CO_2_] is dependent on a variety of fluxes and pools within natural ecosystems. In the following section, we highlight the interactions and trade‐offs between ecosystem cycles. Specifically, we discuss the potential for e[CO_2_]‐induced changes in N availability, increases in site carrying capacity, trade‐offs between plant biomass and soil organic matter accumulation, and changes in the longevity of dead plant C stocks.

### 5.1 Trends in C residence time in dead plant biomass are mixed under e[CO_2_ ]

The residence time of dead plant C constitutes a major determinant of ecosystem C fluxes (Raich & Schlesinger, 1992). Not only does it determine how fast C is released from dead plant biomass, it also defines how long nutrients are locked away in organic tissues and unavailable to plants (Adams et al., 1970). If e[CO_2_]‐induced increases in plant biomass production are coupled with decreased or unchanged decomposition rates, dead plant biomass can be expected to accumulate, retaining both C and nutrients. In contrast, if decomposition rates increase, increased biomass production under e[CO_2_] might correspond to a faster turnover of dead plant C and nutrients. For a full perspective on both terrestrial C sequestration and vegetation trends under e[CO_2_], it is therefore crucial to consider all drivers of C residence time in dead plant biomass. There are several mechanisms by which e[CO_2_] might directly affect decomposition rates of dead organic plant material, including the composition of plant functional types within a community, C allocation patterns within the plant body, and the structural and chemical composition (recalcitrance) of the tissue.

There is considerable variation in the decomposition rates of different plant tissues. In general, non‐woody plant organs decompose faster than woody plant organs (Koehler & Tranvik, 2015; See et al., 2019). Therefore, both a shift in community composition toward woody plant functional types and a change in C allocation patterns toward less readily decomposable plant organs could decrease decomposition rates under e[CO_2_]. The indications of a higher prevalence of woody plant functional types in vegetation communities under e[CO_2_] point toward possible community composition shifts that could decrease ecosystem‐level decomposition rates of dead plant biomass in a high CO_2_ world (see Section 4.2). At the individual plant level, there is contrasting evidence for trends in plant biomass decomposition rates under e[CO_2_]. While older meta‐analyses provide no significant evidence for an increase in root/shoot ratios or higher C allocation to less degradable organs under e[CO_2_] (Curtis & Wang, 1998; Luo et al., 2006), more recent evidence is in line with the expectation that e[CO_2_] aggravates soil resource limitations. Specifically, e[CO_2_] seems to be associated with a considerable increase in root/shoot ratios, with indications of a small increase in the ratio of fine to coarse roots (Nie et al., 2013; Song et al., 2019). As decomposition rates of fine roots are highly variable under different conditions (Berg & McClaugherty, 2008; Zhang & Wang, 2015), it does not seem straightforward how a shift toward higher (fine) root biomass might affect the average C residence time in dead plant tissue under e[CO_2_]. Yet, a higher standing crop of (fine) root biomass in combination with the short lifespan of individual fine roots might cause an increase of C inputs to the soil under e[CO_2_] (Tingey et al., 2000).

Besides a change in C allocation patterns between different tissue types, a change in the chemical composition of plant tissues can also affect decomposition rates of dead plant material. Elevated [CO_2_] generally decrease leaf litter N concentrations and increase levels of leaf litter lignin (Norby et al., 2001), which is indicative of slower decomposition rates (Edmonds & Thomas, 1995). Indeed, leaf litter grown under e[CO_2_] was found to have lower decomposition rates than the control (Norby et al., 2001). However, this trend was driven by low decomposition rates under a single specific CO_2_‐exposure system and turned out to be insignificant without the data from this CO_2_‐exposure system (Norby et al., 2001). In contrast, for root and shoot litter, there were no signs of e[CO_2_]‐induced changes in decomposition rates and—based on three observations only—the same was true for wood litter (Norby et al., 2001). This contradicts observations that wood and roots grown at e[CO_2_] have higher lignin/N and C/N ratios (Cotrufo & Ineson, 2000; Hättenschwiler et al., 1996; Nie et al., 2013), predicting lower decomposition rates (Melillo et al., 1982; Taylor et al., 1989). As similar changes in lignin and N levels were found for leaves without apparent changes in decomposition rates (Norby et al., 2001), this suggests that important mechanisms remain unknown.

While it is often assumed that e[CO_2_]‐induced chemical changes in plant tissue could slow down decomposition rates, emerging research suggests that decomposition under e[CO_2_] might actually be accelerated. In a recent meta‐analysis, the stocks of plant‐derived soil inputs accumulating in mineral soil under e[CO_2_] were found to be stable despite higher soil C inputs as a consequence of e[CO_2_]‐induced increases in biomass production (van Groenigen et al., 2017). Possibly, the higher supply of labile C from root exudates (Phillips et al., 2011) as well as higher soil moisture (Blumenthal et al., 2013; Drake et al., 1997; McCarthy et al., 2010) under e[CO_2_] increased decomposition rates of new soil C (van Groenigen et al., 2017). Overall, the contrasting evidence about rates of plant litter decomposition under e[CO_2_] underscores the need for more long‐term studies. Together with changes in lifecycle dynamics, shifts in tissue decomposition rates underpin how long not only C but also nutrients are stored in the vegetation pool, which emphasizes the importance of quantifying shifts in decomposition rates for evaluating the plant biomass accumulation potential under e[CO_2_].

### 5.2 Plants under e[CO_2_ ] mine soil organic matter for resources needed for increased growth

**FIGURE 5:**
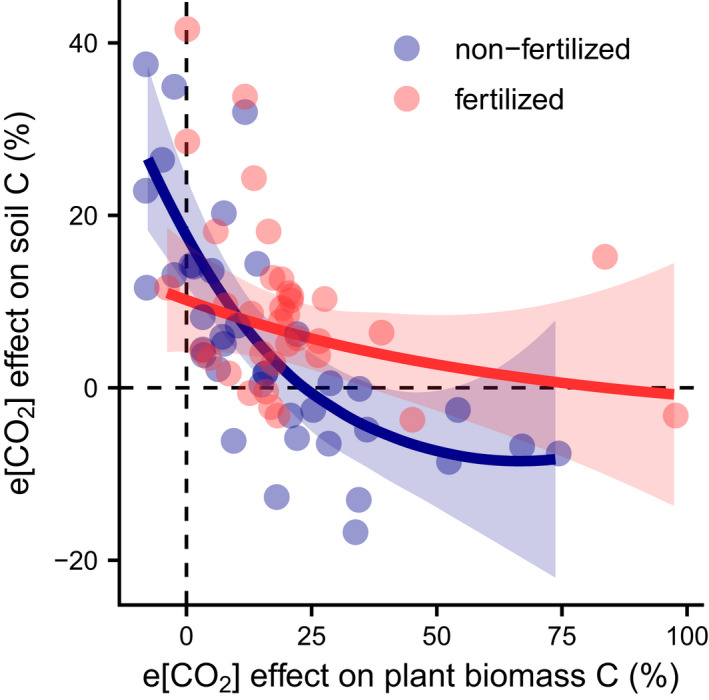
Scatterplot of the e[CO_2_] effect on plant biomass C (%) versus soil organic C (%) in non‐fertilized and N‐fertilized plots. The regression lines are based on a quadratic mixed effects meta‐regression model (non‐fertilized [blue color]: *p* < .0001, *R* ^2^ = .67; fertilized [red]: *p* = .34), in which individual experiments (dots) were weighted differently. The upper and lower limits of the 95% confidence interval are indicated by the light ribbons. Data extracted from Terrer et al. (2021) using WebPlotDigitizer (Rohatgi, 2021).

Community‐level evidence suggests that plants associating with specific mycorrhiza might be able to sustain e[CO_2_]‐induced plant growth stimulation under N‐limited conditions. In this context, EMF have been shown to alleviate N limitations in their symbiotic plant hosts by acquiring additional nutrients from the soil (Terrer et al., 2016; see Section 4.1). This sets up the expectation for a trade‐off between the plant and soil pool in nutrient‐limited ecosystems under e[CO_2_]. Yet, this is in direct conflict with the common assumption that increased inputs of dead plant tissue to the soil resulting from higher plant growth rates under e[CO_2_] will increase the soil organic C (SOC) pool. This relationship is a primary assumption embedded in most ESMs (Todd‐Brown et al., 2014). However, a recent meta‐analysis (Terrer et al., 2021) of temperate‐zone CO_2_ experiments reported a negative relationship between plant biomass and SOC accumulation under e[CO_2_], whereby SOC increased slightly when e[CO_2_] caused a weak plant biomass increase but decreased when e[CO_2_] strongly stimulated plant biomass accumulation (Figure 5). This trend was only significant in non‐fertilized but not in N‐fertilized systems. In unfertilized systems, SOC losses coupled with strong biomass gains were usually measured for species associated with EMF. This suggests that the observed effect might be driven by nutrient acquisition processes such as priming (Kuzyakov, 2002), where N‐limited plants afford biomass production by actively stimulating microbial decomposition via ectomycorrhizal symbioses (Terrer et al., 2021). In contrast, AMF‐associated plants in unfertilized systems did not show a strong biomass response to e[CO_2_] while SOC was stimulated (Terrer et al., 2021), likely due to increased C inputs through higher fine root production and rhizodeposition (Nie et al., 2013). This evidence from temperate ecosystems for a trade‐off between plant and soil C suggests that mycorrhizal associations of certain plant functional types and species can help to sustain e[CO_2_]‐induced plant biomass accumulation even under N limitation.

### 5.3 N cycle adaptations under e[CO_2_ ] might slightly alleviate progressive N limitation

Multiple studies have indicated the need for incorporating N limitation of the plant biomass response to e[CO_2_] in global vegetation models (Davies‐Barnard et al., 2020; Wieder et al., 2015). Indeed, at each level of ecological scale highlighted in this review, we find compelling evidence that the degree of C uptake and plant growth under e[CO_2_] is strongly dependent on ecosystem N fluxes. While many CO_2_ enrichment studies have focused on different plant traits and strategies to either mine N or cope with N limitation under e[CO_2_], there is less commonly a focus on variations in N availability under changing [CO_2_]. However, while an increase in standing biomass also increases the amount of N that is locked up in biomass (PNL; Comins & McMurtrie, 1993; Luo et al., 2004), e[CO_2_] may also influence N retention over time. The amount of plant‐available N is determined both by the input and output of N in an ecosystem as well as the mineralization of the given N stocks. It could be expected that N mineralization increases under e[CO_2_] because of e[CO_2_]‐induced increases in soil moisture (Blumenthal et al., 2013; Drake et al., 1997; McCarthy et al., 2010) and the amount of C entering the soil (Finzi et al., 2007; Norby et al., 2004). However, there is consistent evidence from multiple meta‐analyses that neither gross nor net N mineralization rates are sensitive to e[CO_2_] (de Graaff et al., 2006; Liang et al., 2016; Rütting & Andresen, 2015). Yet, the finding that e[CO_2_] stimulates gross N mineralization in N‐ but not P‐limited ecosystems implies that N‐limited ecosystems might be less prone to PNL than currently assumed (Rütting & Andresen, 2015), which could be studied through ecosystem‐specific analyses of net N mineralization rates.

Apart from changes in N mineralization rates, changes in N availability could occur through changes in the ecosystem balance of N inputs and outputs. Higher N input through biological fixation would be intuitive under e[CO_2_] as plants that have access to more N could have a competitive advantage (see Section 4.1). Indeed, there is an increase in N fixation under e[CO_2_] (Liang et al., 2016), possibly due to both higher activity of N‐fixing bacteria (Hoque et al., 2001) and competitive selection for N‐fixing species (Batterman et al., 2013). This increase in NH_4_^+^ input is accompanied by decreased leaching under e[CO_2_] (Liang et al., 2016), likely as a result of lower levels of NO_3_^‐^, which is the form of N most prone to leaching (Barnard et al., 2005), and higher root biomass under e[CO_2_] (Liang et al., 2016; Nie et al., 2013; Song et al., 2019). By contrast, increases in N losses (N_2_O) were less pronounced and only occurred in studies with N addition or upland soils (Liang et al., 2016; van Groenigen et al., 2011). Together with increases in N fixation and lower leaching, there may thus be an alleviation of PNL under e[CO_2_] in many ecosystems. While it is unlikely that these N cycle adaptations can prevent N limitation under e[CO_2_] (Norby et al., 2010; Reich & Hobbie, 2013; see Figure 3), even small changes in N dynamics can have a considerable impact at the large scale and should therefore be considered in ESMs.

### 5.4 Trends in site carrying capacity under e[CO_2_ ] are unclear

Every ecosystem can only sustain a certain amount of biomass due to a limited availability of resources such as light, space, nutrients, and water. However, this does not necessarily mean that the maximum amount of plant biomass in an ecosystem is a constant. Climate‐driven shifts in the composition or traits of plants in a community can drastically alter carrying capacities, especially if they are associated with major functional group shifts. However, along with shifts from non‐woody to woody functional groups, shifts within functional groups can also increase C storage potential and the residence time of nutrients in plant biomass. In the only study we found on this topic in the context of plant biomass responses to e[CO_2_], a North American temperate broadleaf ecosystem growing under e[CO_2_] followed self‐thinning curves of stands with a higher site carrying capacity (Kubiske et al., 2019). The authors suggest that this result might be owed to increased resource‐use efficiency or resource availability under e[CO_2_], both of which we have previously discussed (see Sections 2.1, 4.1, 5.3). Yet, emerging evidence across multiple ecological scales suggests that biomass responses to e[CO_2_] might dampen over time (see Sections 3.1, 3.2, 4.1). These mixed results may indicate that the effect of e[CO_2_] on site carrying capacity might be context‐dependent and more, long‐term research is required.

## 6 GLOBAL ECOLOGY

Global‐scale studies provide the opportunity to map the effects of rising [CO_2_] over the past decades and extrapolate results from predominantly short‐term physiological‐ to community‐scale studies to project the long‐term impacts of e[CO_2_] on global vegetation biomass. While this large‐scale view provides insight to the potential effects of e[CO_2_] at the global scale, it also glosses over many of the details discussed in previous sections. Below we summarize the key findings from global satellite imagery and extrapolations from CO_2_ enrichment studies. We highlight commonalities and discrepancies between global‐scale patterns and lower levels of ecological organization (physiology to ecosystem scale), with the explicit aim of motivating future research that is needed to improve the accuracy of future climate change projections.

### 6.1 Global greening trend over last decades might have weakened

**FIGURE 6:**
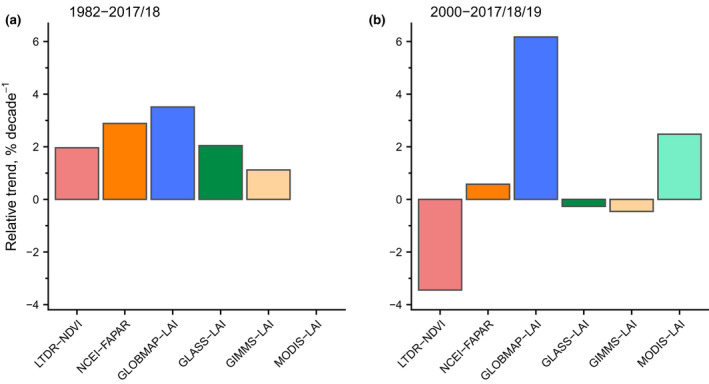
Global leaf area trends in six different remote sensing datasets. Displayed are the average changes (% decade^−1^) during 1982–2017/2018 (a) and 2000–2017/2018/2019 (b). Data extracted from Winkler et al. (2021) using WebPlotDigitizer (Rohatgi, 2021).

Since atmospheric [CO_2_] have been increasing since the beginning of industrialization, the analysis of historic satellite imagery provides an opportunity to evaluate the effect of e[CO_2_] on plant biomass from another angle than CO_2_ enrichment studies. As e[CO_2_] increases plant WUE, warm and arid ecosystems are expected to show the strongest plant response to e[CO_2_] (see Section 2.1). Indeed, between 1982 and 2010, green foliage cover (measured as normalized difference vegetation index) in such ecosystems has increased by 11% and gas exchange theory suggests that this trend was largely driven by e[CO_2_] (Donohue et al., 2013). Multiple satellite studies have reported global greening since the 1980s, namely an increase in tree cover (7.1%; Song et al., 2018), higher leaf area index (LAI; 2.3% dec^−1^: Chen et al., 2019; 0.068 m^2^ m^−2^ year^−1^: Zhu, Piao, et al., 2016) and/or less bare ground cover (−3.1%; Song et al., 2018). Yet, as multiple parameters have changed over the last decades, the effect of e[CO_2_] needs to be disentangled from those of changes in precipitation, temperature, or N deposition. While one of the mentioned greening studies (Zhu, Piao, et al., 2016) estimates that 70% of the observed increase in LAI can be attributed to increasing [CO_2_], more recent findings suggest that e[CO_2_] might not be a dominant driver of greening in many ecosystems (Winkler et al., 2021). While the effect of e[CO_2_] on plant biomass emerged as the main driver of greening in some biomes (temperate forests, cool grasslands, likely also Australian shrublands), the greening and browning (decrease in vegetation cover) trends of many other regions seemed to be linked to climate change, primarily warming and drying (Winkler et al., 2021). In general, there are indications that the widely observed greening trend might be slowing down and more areas seem to show decreases in LAI, possibly resulting in a negative global biomass trend for the period 2000–2017/2018/2019 (Winkler et al., 2021; Figure 6). These findings might either suggest that the effect of e[CO_2_] on plant biomass is dampening or that it is weighed out by other global trends. Recent evidence supports the first of these two options by suggesting that satellite data estimates of e[CO_2_]‐induced photosynthetic stimulation since the 1980s have declined at considerably higher rates relative to estimates from terrestrial C cycle models (0.92% vs 0.12% per 100 ppm CO_2_), potentially due to model algorithms not adequately considering emerging nutrient limitation constraints and the effect of changing water availability on the plant response to e[CO_2_] (Wang et al., 2020). Yet, these results have been questioned due to the selection and processing of the datasets (Frankenberg et al., 2021; Sang et al., 2021; Wang et al., 2021; Zhu et al., 2021). Conclusively, the presented evidence underlines the difficulty of isolating the effect of e[CO_2_] from long‐term observational data, and while there seems to be agreement of global‐scale studies on e[CO_2_]‐induced biomass accumulation, evidence may or may not suggest a dampening of the effect of e[CO_2_] on plant biomass over the last decades.

### 6.2 Extrapolation of CO_2_ enrichment studies suggests e[CO_2_ ]‐induced biomass increases

The average effect size in CO_2_ enrichment studies might not be a good proxy for the global biomass response to e[CO_2_] due to the fact that experiments in Northern Hemisphere ecosystems with relatively fertile soils are overrepresented (Terrer et al., 2019). Therefore, an extrapolation approach considering the global patterns of drivers of the plant biomass response to e[CO_2_] is the most direct way to use CO_2_ enrichment studies for a global estimate of vegetation responses to e[CO_2_]. Such an analysis using global layers of soil C:N, P, and mycorrhizal type suggests that an increase in the current [CO_2_] by 250 ppm as expected for 2100 will enhance global plant biomass by 12%, which corresponds to 5% per 100 ppm CO_2_ (Terrer et al., 2019). A comparison with the more than three times higher average estimate of nine terrestrial C cycle models for the period of 1980–2010 (18% per 100 ppm CO_2_) indicates a decrease in the effect of e[CO_2_] on plant biomass over time (Terrer et al., 2019). With the effect of possible changes in lifecycles of perennial plants widely excluded due to the short duration of most CO_2_ enrichment studies, this result is consistent with the expectation that the depletion of soil resources might prevent a widespread sustained stimulation of biomass under e[CO_2_] over time.

## 7 CONCLUSIONS AND RECOMMENDATIONS

The effect of e[CO_2_] on plant biomass stocks is shaped by processes that occur across all scales from cells to ecosystems. Adding to a traditional focus on e[CO_2_]‐induced stimulations of photosynthesis and plant growth, recent work reveals a suite of mechanisms operating at the level of individuals, populations, communities, and ecosystems that are expected to drive vegetation trends in a high‐CO_2_ environment. Yet, before this can most effectively inform ESMs, a reconciliation of evidence from the different scales is needed. While there might be considerable cross‐scale interactive modulation of e[CO_2_] impacts by climate change itself (i.e., amount and timing of changes in temperature, precipitation, etc.; Reich et al., 2020; Yuan et al., 2018; Zhu, Chiariello, et al., 2016), which is beyond the scope of this review, the presented evidence on direct effects of e[CO_2_] on plant biomass offers important indications for the expected response of terrestrial ecosystems to global change.

We find that the evidence for a sustained biomass response to e[CO_2_] varies across ecological scales. At the level of physiology, e[CO_2_]‐driven increases in WUE and the operational capacity of Rubisco are expected to drive a long‐term stimulation of photosynthesis under e[CO_2_] despite photosynthetic acclimation. At the population level, evidence suggests that plant growth increases under e[CO_2_] may dampen over time in low N and P conditions and that earlier life stages may be more responsive than later life stages. In addition, there remains considerable uncertainty around changes in other demographic processes, specifically mortality, that have the potential to counterbalance the effect of plant growth rate increases and lead to a dampening effect of e[CO_2_] on plant biomass accumulation over time. Evidence at the scale of community ecology suggests that e[CO_2_] can alter competitive dynamics among species within plant communities. For example, if different plant responses to e[CO_2_] lead to the encroachment of woody plants relative to herbaceous species, this could lead to increases in ecosystem carrying capacity and total C storage. At the ecosystem‐scale, there might be indications for e[CO_2_]‐driven changes in resource pools and fluxes, which might potentially support increased plant growth rates under e[CO_2_]. Yet, possible trade‐offs between plant and soil C might suggest that a sustained effect of e[CO_2_] on plant biomass levels might not necessarily equate with an increase in long‐term C sequestration at the entire ecosystem scale. At the global scale, global greening might suggest that the aggregate of all of the finer‐scale mechanisms tends toward increasing plant C levels under e[CO_2_]. Yet, the fact that this trend might dampen and the low signal to noise ratio introduces further uncertainty that restricts confidence in future projections. Since the research focus varies across different ecological scales, it becomes evident that only an integration of research across ecological scales can yield a complete picture of the potential plant biomass responses to e[CO_2_].

To improve the accuracy of global C cycle predictions, it becomes increasingly clear that research must focus on integrating the key mechanisms that occur across all ecological scales to facilitate the projection of short‐term results into the future. Given a vast array of different mechanisms operating within individual plants, populations, communities, and ecosystems, modeling efforts will require considerable sensitivity testing to identify which processes are most important to represent. In addition, it is crucial to consider the connectedness of the plant biomass pool with other biotic and abiotic pools of C and nutrients. By applying this wide focus in models and experiments about the effect of e[CO_2_] on plant biomass and C sequestration, we can move forward across scales to narrow this key uncertainty in our understanding of climate change.

## AUTHOR CONTRIBUTIONS

JM, TWC (Ph.D. advisor), and LBM (second Ph.D. advisor) conceived the paper. JM performed the data analysis with the help of LBM and JW. JM drafted the article with major contributions from LBM and TWC; all other authors also assisted with writing the manuscript. LCA, MCB, PBR, AL, MKS, CM, GM, JSD, KZ, and IKS contributed data. All authors approved the final version to be published.

## Supporting information


Appendix S1

## Data Availability

The data that support the findings of this study are openly available in Dryad at https://doi.org/10.5061/dryad.hhmgqnkk4.
