# Supplementary material for: Links across ecological scales: Plant biomass responses to elevated CO2
Source: Glob Chang Biol. 2022 Sep 7;28(21):6115–34. doi: 10.1111/gcb.16351 (PMC9825951; doi:10.1111/gcb.16351)
Supplement: Supplementary file 1 — Appendix S1 [file GCB-28-6115-s001.docx]

# Supplements

## 1. Supplementary Methods

### 1.1 Data sources and construction

The data used for our analyses were adapted from Andresen et al. (2016). This dataset contained treatment-level aboveground standing biomass (non-trees) and annual aboveground biomass increment data (trees) from CO_2_ manipulation experiments (Supplementary Table 1). For our analyses, we used all data comparing ambient to elevated CO_2_ concentrations (a[CO_2_], e[CO_2_]), including cases where other treatments had been applied to both CO_2_ levels (e.g. a[CO_2_]-warming vs e[CO_2_]-warming) (Supplementary Table 1). In total, our subset had 331 observations from nine different experimental sites. We did not carry out corrections for pre-treatment differences because we only had pre-treatment values from five of the nine experimental sites. In R version 4.2.1 (R Core Team, 2022), we fit mixed effect linear models using the lmer function from the lme4 package (Bates et al., 2015) and obtained *P* values and estimates for model coefficients using the lmerTest package (Kuznetsova et al., 2017) and the summary function. We obtained marginal (variance explained by fixed effects) and conditional R^2^ (variance explained by entire model) using the r.squaredGLMM function from the MuMIn package (Bartoń, 2022). Like in Andresen et al. (2016), the response variable was ln(aboveground biomass under e[CO_2_]) – ln(aboveground biomass under a[CO_2_]) where aboveground biomass means aboveground standing biomass for non-trees and a measure of annual aboveground biomass increment for trees.

To characterize the effects of nitrogen on plant biomass responses to e[CO_2_] over time, we assigned all observations to one of two nitrogen treatments (low N, high N), depending on if N fertilizer had been added or not. The only exception to this was ETH Swiss FACE, where not only the high N treatments were fertilized but also the low N treatments received small amounts of N fertilizer. We assigned these low N treatments to our low N group; however, assigning it to high N did not change the directionality or significance of the predictor variables. Similarly, excluding the observations of N fixing species (*Trifolium repens* at ETH Swiss FACE) did not affect our results (except for that model selection dropped the random effect of *experiment* while keeping the nested random effect, see details about variables below). We only used observations with non-tree functional types for this analysis because there were no high N observations for trees in the dataset.

To enable the analysis of differences in e[CO_2_]-induced plant biomass responses between functional types, we assigned each observation to the dominant plant functional type (tree, non-tree). We excluded the high N treatment observations from this analysis because this treatment had not been done for trees. While aboveground standing biomass was measured for non-trees and annual increment in aboveground plant biomass was measured for trees, we considered these values to be comparable because aboveground tree biomass is a cumulative measure and we were primarily interested in characterizing how the annual rate of plant biomass production changed over time.

### 1.2 Data analysis

#### 1.2.1 Effect of nitrogen on plant biomass response to e[CO_2_]

To test for the effects of nitrogen and year on the plant biomass response to e[CO_2_], we first constructed a second-order polynomial regression model that included the main effects of *nitrogen* (as an integer: low N = 0, high N = 1), *time*, and *time^2^* as well as the product of *nitrogen* and each of the two main effects *time* and *time^2^*. To control for non-random differences in e[CO_2_] plant responses (e.g., warming and drought treatments, see Supplementary Table 1), we included *experiment* as well as *additional treatment* nested within *experiment* as random factors. Specifically, *additional treatment* refers to non-CO_2_ and non-N treatments, i.e., different species assemblages, water, or temperature regimes, at the same experimental site which were identical in the low- and high-CO_2_ treatment. To retain all potentially important terms in our model, we conducted stepwise backward model selection using the step function from the lmerTest package, which uses F-tests for fixed effects and likelihood ratio tests for random effects. During variable selection, we allowed main effects to be dropped even when their products or quadratic polynomial terms were retained as the resulting model would still be interpretable (see below). The best supported model included *time^2^* as a main effect, the product of *nitrogen* and *time^2^*, and the two random effects (*experiment*, *additional treatment* nested within *experiment)*. The main effect of *nitrogen* being dropped from the best supported model indicated that, at the start of the experiment, there was no difference in the biomass response to e[CO_2_] between the two nitrogen treatments. This implies that the dampening effect of N limitations on the e[CO_2_] effect developed over time. The fact that *time* and *time*nitrogen* were excluded from the best supported model suggests that, at the start of the experiment, the slopes of the biomass response to e[CO_2_] in the low and high N treatments, respectively, were zero.

We tested if model terms were significantly different from zero using the lmerTest package and the summary function (*P* values: *time^2^* = 0.025, *nitrogen*time^2^* = 0.003). Model results and data are presented in Supplementary Table 2, Figure 3 (excl. pre-treatment values), Supplementary Figure 1 (incl. pre-treatment values) and Supplementary Figure 2 (time series for each experiment). Using model-based bootstrapping, we calculated model predictions and approximated the 95% confidence interval, using the bootMer function from the lme4 package. Specifically, we ran 1000 simulations and obtained the upper and lower limits of the confidence intervals by adding/subtracting the standard error (estimated as the standard deviation of the bootstrap iterations) times 1.96 to/from the predicted value.

For model construction, we considered data over the complete range of time in the dataset, i.e. years 1–18, as there were both high and low N observations for each year. Due to the different durations of the used studies, this meant that for the last two years we were only relying on data from two time series (see Supplementary Figure 2). However, running the analysis with data from years 1–16 (data from eleven time series in the last year) did not change the directionality or significance of predictors in the best model except for the fact that the model selection function kept the main effect of *time* for the final model (estimate = 2.081e-02, *P* = 0.036) and the estimate of the intercept became negative (-2.840e-04 vs 6.288e-02). The best model with data from years 1–10 (data from 15 time series in last year) contained only a negative intercept (estimate = -0.003, *P* = 0.944), the main effect of *time* (estimate = 0.012, *P* = 0.004) and the two random effects. This illustrates that, in our dataset, N limitation in the low N treatment manifested only after more than a decade. Model diagnostics plots for the years 1–18 were created with the residplot function from the predictmeans package (Luo et al., 2021) and are displayed in Supplementary Figure 3.

#### 1.2.2 Effect of functional type on plant biomass response to e[CO_2_]

To test for significant differences between functional groups over time, we first constructed a second-order polynomial model, which – fully parameterized – included the main effects of *functional type* (as an integer: non-tree = 0, tree = 1), *time*, and *time^2^*, the products of *functional type* and each of the two main effects *time* and *time^2^* as well as the two random effects that were also in the N model (*experiment*, *additional treatment* nested within *experiment*). To retain only potentially important terms in our model, we conducted stepwise backward model selection. Again, we allowed main effects to be dropped even when their products or quadratic polynomial terms were retained as the resulting model would still be interpretable. The best supported model included the main effects *time* and *time^2^* as well as the two random effects. In the last iteration of the stepwise model selection process, the term *functional type* was dropped. However, our power to detect significant differences between functional types was reduced as three of the five times series from tree experiments in our dataset where from the same experimental site (Aspen FACE) and thereby non-independent. Also, the term *functional type* became significant (*P* = 0.041) when we removed the observations from N fixing species (*Trifolium repens* at ETH Swiss FACE). Thus, to explore general trends among functional types, we retained the main effect of *functional type* in our final model (we also kept the N fixer data). Using the lmerTest package and summary on the model including *functional type*, we found that the main effects of *time* and *time^2^* were significantly different from zero (both *P* values < 0.00006) while *functional type* had a marginally significant *P* value (*P* = 0.051). Model results and data are presented in Supplementary Table 2, Figure 4 (excl. pre-treatment values), Supplementary Figure 4 (incl. pre-treatment values) and Supplementary Figure 5 (time series across each experiment). Using model-based bootstrapping, we calculated model predictions and the approximated confidence intervals, using the bootMer function from the lme4 package. For model construction, we considered data from the period where we had both tree and non-tree observations, i.e. years 1–12. Model diagnostics plots were created with the residplot function and are displayed in Supplementary Figure 6.

## 2. Supplementary Tables

| **Experiment** | **Treatment** | **Functional Type** |
| --- | --- | --- |
| Aspen FACE (US) | a[CO_2_] vs e[CO_2_] aspen  a[CO_2_] vs e[CO_2_] aspen and birch  a[CO_2_] vs e[CO_2_] aspen and maple | tree |
| BioCON FACE (US) | a[CO_2_] vs e[CO_2_]  a[CO_2_]-N vs e[CO_2_]-N | non-tree |
| CLIMAITE (DK) | a[CO_2_] vs e[CO_2_]  a[CO_2_]-drought vs e[CO_2_]-drought  a[CO_2_]-warming vs e[CO_2_]-warming  a[CO_2_]-drought&warming vs e[CO_2_]-drought&warming | non-tree |
| Duke FACE (US) | a[CO_2_] vs e[CO_2_] | tree |
| ETH Swiss FACE (CH) | a[CO_2_]-lowN vs e[CO_2_]-lowN *Lolium perenne*  a[CO_2_]-highN vs e[CO_2_]-highN *Lolium perenne*  a[CO_2_]-lowN vs e[CO_2_]-lowN *Trifolium repens*  a[CO_2_]-highN vs e[CO_2_]-highN *Trifolium repens* | non-tree |
| GI-FACE (GER) | a[CO_2_]-N vs e[CO_2_]-N | non-tree |
| Jasper Ridge FACE (US) | a[CO_2_] vs e[CO_2_]  a[CO_2_]-N vs e[CO_2_]-N  a[CO_2_]-N-warming vs e[CO_2_]-N-warming  a[CO_2_]-warming vs e[CO_2_]-warming  a[CO_2_]-N-warming-water vs e[CO_2_]-N-warming-water  a[CO_2_]-warming-water vs e[CO_2_]-warming-water  a[CO_2_]-N-water vs e[CO_2_]-N-water  a[CO_2_]-water vs e[CO_2_]-water | non-tree |
| Oak Ridge FACE (ORNL) (US) | a[CO_2_] vs e[CO_2_] | tree |
| PHACE (US) | a[CO_2_] vs e[CO_2_]  a[CO_2_]-warming vs e[CO_2_]-warming | non-tree |

Supplementary Table 1 | Overview of dataset (treatments, functional types). Treatments in orange are additional treatments embedded in the models as nested random effects within experiment.

| **Model** | **Predictor** | **Estimate** | **df** | ***P*** | **R^2^** |
| --- | --- | --- | --- | --- | --- |
| nitrogen | intercept | 6.288e-02 | 6.174 | 0.128 | 0.03 (0.21) |
|  | time^2^ | -4.117e-04 | 256.0 | **0.025** |  |
|  | nitrogen*time^2^ | 5.990e-04 | 255.8 | **0.003** |  |
| funct. type (1) | intercept | -0.007 | 17.104 | 0.905 | 0.05 (0.55) |
|  | time | 0.056 | 154.625 | **5.67e-05** |  |
|  | time^2^ | -0.005 | 154.783 | **1.68e-05** |  |
| funct. type (2) | intercept | -0.077 | 12.625 | 0.216 | 0.24 (0.54) |
|  | time | 0.056 | 154.800 | **5.42e-05** |  |
|  | funct. type | 0.196 | 6.326 | 0.051 |  |
|  | time^2^ | -0.005 | 155.065 | **1.65e-05** |  |

Supplementary Table 2 | Estimated coefficients for the reduced models. From the analysis of the effect of functional type, we present the coefficients of both the model from stepwise model selection (excluding the variable functional type) as well as of the reduced model with the variable functional type added. df = degrees of freedom, R^2^ presented as marginal R^2^ (conditional R^2^), P values significant at the 5% level are marked in bold, ”*” denotes a multiplication of main effects.

## 3. Supplementary Figures

Supplementary Figure 1 | Effect of N addition on the plant biomass response to e[CO_2_] over time (incl. pre-treatment). Points are colored according to the N treatment (low N = pink, high N = blue). In the same color code, we added predictions and approximated 95% confidence intervals. The model was calculated based on all treatment years (i.e., 1–18). Years of exposure before 1 are pre-treatment values. Pre-treatment points are colored according to the N regime during the CO_2_ treatment, i.e., are also depicted as “high N” during pre-treatment years if the N treatment only started at year 1. Plots without pre-treatment year data, time series of individual experiments and model diagnostics are visualized in Figure 3 and Supplementary Figures 2 and 3.

Supplementary Figure 2 | Time series of the experiments and treatments used to model the effect of N addition on the plant biomass response to e[CO_2_] over time (incl. pre-treatment). Points and lines are colored according to the experimental site and treatment (species, water, temperature). Vertical dashed lines mark the time span of the data that was used for modeling the effect of N addition on the plant biomass response to e[CO_2_] over time. The model is plotted out in Figure 3 and Supplementary Figure 1.

Supplementary Figure 3 | Diagnostics plots for the model of the effect of N addition on the plant biomass response to e[CO_2_].

Supplementary Figure 4 | Effect of functional type on the plant biomass response to e[CO_2_] over time (incl. pre-treatment). Points are colored according to the dominant plant functional type (green = tree, yellow = non-tree). In the same color code, we added predictions and approximated 95% confidence intervals. The model was calculated based on treatment years shared between both functional type groups (i.e., 1–12). Years of exposure before 1 are pre-treatment values. Plots without pre-treatment year data, time series of individual experiments and model diagnostics are visualized in Figure 4 and Supplementary Figure 5 and 6.

Supplementary Figure 5 | Time series of the experiments and treatments used to model the effect of functional type on the plant biomass response to e[CO_2_] over time (incl. pre-treatment). Points and lines are colored according to the experimental site and treatment (species, water, temperature). Vertical dashed lines mark the time span of the data that was used for modeling the effect of functional type on the plant biomass response to e[CO_2_] over time. The model is plotted out in Figure 4 and Supplementary Figure 4.

Supplementary Figure 6 | Diagnostics plots for the model of the effect of functional type on the plant biomass response to e[CO_2_] (model “funct. type (2)” in Supplementary Table 2).

## 4. Supplementary Bibliography

Andresen, L. C., Müller, C., de Dato, G., Dukes, J. S., Emmett, B. A., Estiarte, M., et al. (2016). “Shifting Impacts of Climate Change: Long-Term Patterns of Plant Response to Elevated CO2, Drought, and Warming Across Ecosystems,” in *Advances in Ecological Research* doi:10.1016/bs.aecr.2016.07.001.

Bartoń, K. (2022). MuMIn: Multi-Model Inference. Available at: https://cran.r-project.org/package=MuMIn.

Bates, D., Maechler, M., Bolker, B., and Walker, S. (2015). Fitting Linear Mixed-Effects Models Using lme4. *J. Stat. Softw.* 67, 1–48. doi:10.18637/jss.v067.i01.

Kuznetsova, A., Brockhoff, P. B., and Christensen, R. H. (2017). lmerTest Package: Tests in Linear Mixed Effects Models. *J. Stat. Softw.* 82, 1–26. doi:10.18637/jss.v082.i13.

Luo, D., Ganesh, S., and Koolaard, J. (2021). predictmeans: Calculate Predicted Means for Linear Models. Available at: https://cran.r-project.org/package=predictmeans.

R Core Team (2022). R: A language and environment for statistical computing. Available at: https://www.r-project.org/.
